# Tertiary lymphoid structures sustain cutaneous B cell activity in hidradenitis suppurativa

**DOI:** 10.1172/jci.insight.169870

**Published:** 2024-02-08

**Authors:** Margaret M. Lowe, Jarish N. Cohen, Madison I. Moss, Sean Clancy, James P. Adler, Ashley E. Yates, Haley B. Naik, Rashi Yadav, Mariela Pauli, Ian Taylor, Austin McKay, Hobart Harris, Esther Kim, Scott L. Hansen, Michael D. Rosenblum, Joshua M. Moreau

**Affiliations:** 1 Department of Dermatology, University of California, San Francisco (UCSF), San Francisco, California, USA.; 2 Bioinformatics and Genomics master’s program, University of Oregon, Eugene, Oregon, USA.; 3 Cancer Early Detection Advanced Research Center, Oregon Health & Science University (OHSU), Portland, Oregon, USA.; 4 TRex Bio, South San Francisco, California, USA.; 5 Department of Surgery, UCSF, San Francisco, California, USA.; 6 Division of Oncological Sciences,; 7 Department of Dermatology, and; 8 Department of Cell, Developmental and Cancer Biology, OHSU, Portland, Oregon, USA.

**Keywords:** Dermatology, Immunology, Adaptive immunity, Skin

## Abstract

Hidradenitis suppurativa (HS) is a chronic skin condition affecting approximately 1% of the US population. HS skin lesions are highly inflammatory and characterized by a large immune infiltrate. While B cells and plasma cells comprise a major component of this immune milieu, the biology and the contribution of these cells in HS pathogenesis are unclear. We aimed to investigate the dynamics and microenvironmental interactions of B cells within cutaneous HS lesions. Combining histological analysis, single-cell RNA sequencing, and spatial transcriptomics profiling of HS lesions, we defined the tissue microenvironment relative to B cell activity within this disease. Our findings identified tertiary lymphoid structures (TLSs) within HS lesions and described organized interactions among T cells, B cells, antigen-presenting cells, and skin stroma. We found evidence that B cells within HS TLSs actively underwent maturation, including participation in germinal center reactions and class switch recombination. Moreover, skin stroma and accumulating T cells were primed to support the formation of TLSs and facilitate B cell recruitment during HS. Our data definitively demonstrated the presence of TLSs in lesional HS skin and point to ongoing cutaneous B cell maturation through class switch recombination and affinity maturation during disease progression in this inflamed nonlymphoid tissue.

## Abstract 2

<p>Single cell RNA sequencing and spatial transcriptomics reveal ongoing B cell maturation in lesional skin of hidradenitis suppurativa patients.</p>

## Graphical abstract

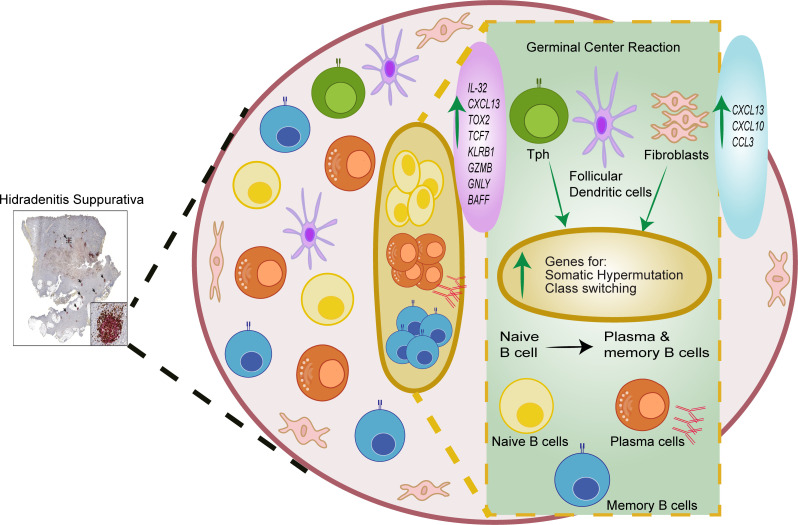

## Introduction

Hidradenitis suppurativa (HS) is a chronic inflammatory skin condition primarily occurring in intertriginous regions of the body affecting approximately 1% of the US population (1). HS may progress from acne-like inflammatory nodules to recurrent abscesses, formation of extensive sinus tracts, and deep-seated fibrosis and scarring (1). Treatment strategies for HS include antimicrobial washes and antibiotics, steroids, hormonal therapies, antiinflammatory biologics, and surgery to resect areas of advanced fibrosis. Targeted biologic treatments, such as anti–TNF-α agents, however, do not provide clinical benefit to all patients, which may be linked to the extent of immune activation within the skin before treatment (1). A more detailed understanding of the immune pathogenesis in HS is required to catalyze development of effective targeted therapies.

Immune dysregulation surrounding the hair follicle following follicular occlusion is hypothesized to initiate the aberrant inflammatory process characteristic of HS plaques; however, exact disease etiology is unclear (1–3). For example, immune infiltrates within HS lesions include overrepresentation of cell subsets that are rarely observed in healthy skin and other inflammatory skin diseases, especially B cells and plasma cells (4–7). In addition, antibodies recognizing a broad array of autoantigens have been identified in patient sera as well as lesional skin, suggesting contribution of B lineage cells to disease pathogenesis (6–11). Limited studies involving B cell depletion with rituximab hint that modulation of these cells may have therapeutic benefit (12, 13). Lymphoid aggregates comprising B cells and T cells have been described in HS lesions and are a recognized histological feature of this disease, but it is unknown if organized tertiary lymphoid structures (TLSs) capable of sustaining cutaneous B cell activity in situ are present (1, 4, 7, 14). To understand the dynamics and microenvironmental interactions of B cells within HS lesions, we employed histological analysis, single-cell RNA sequencing (scRNA-Seq), and spatial transcriptomics. Our data definitively demonstrate the presence of functional TLSs in this disease and define the molecular identity and contributing stromal niche for generation of these structures in diseased HS skin.

## Results

### TLSs are present in HS skin.

**Figure 1:**
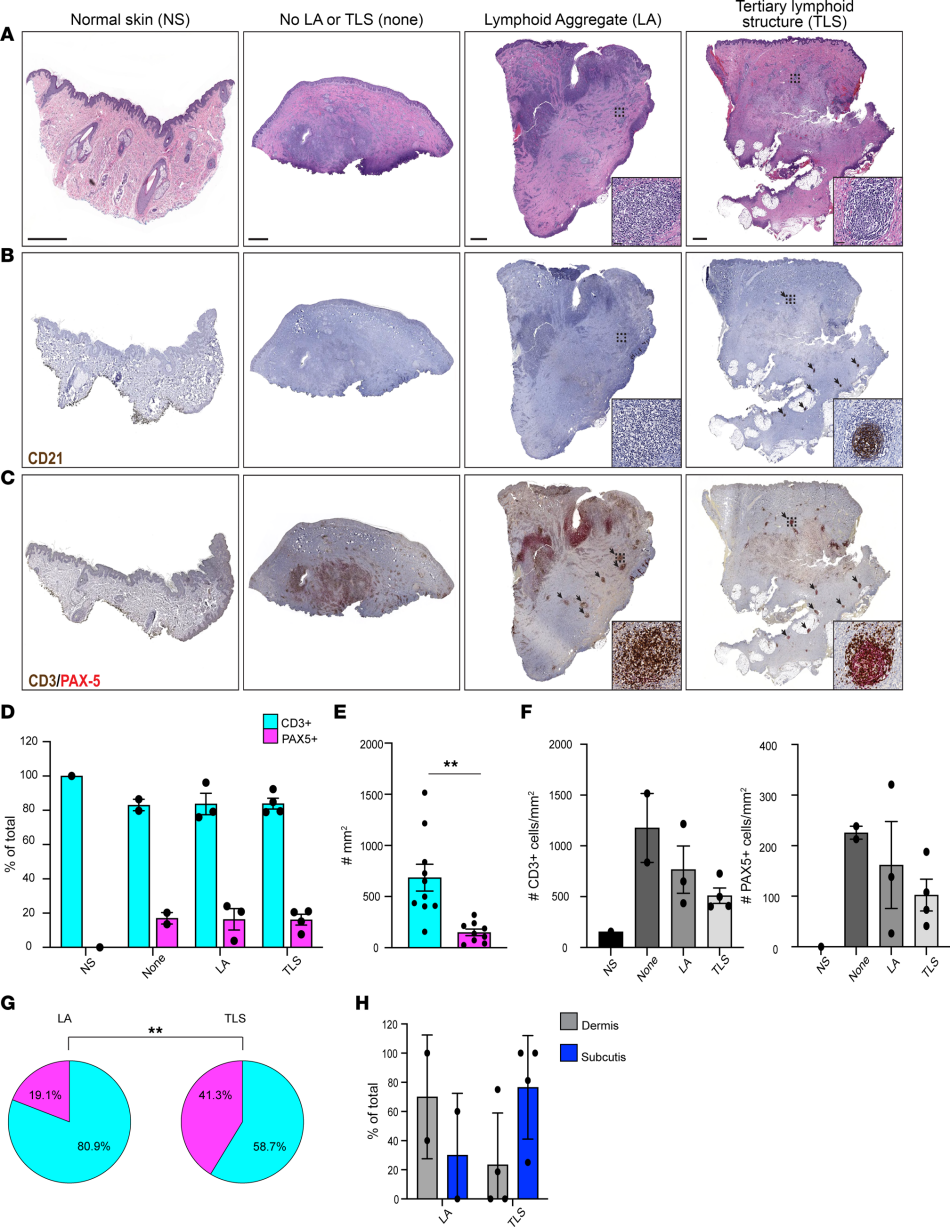
TLSs are present in HS skin. (**A**) Low-power photomicrographs of H&E-stained sections of healthy and HS skin with the indicated features. Dashed boxes indicate representative LAs or TLSs, and insets show a higher power magnification image within dashed boxes; scale bars = 2 mm. (**B**) Low- and higher power (insets) photomicrographs of CD21 immunohistochemistry (IHC) on normal and HS skin. (**C**) Low- and higher power (insets) photomicrographs of multiplex CD3 (brown chromogen)/PAX5 (red chromogen) IHC on normal and HS skin. Black arrows indicate LAs and TLSs. PAX5, paired box protein 5. (**D**) Frequency of CD3^+^PAX5^–^ and CD3^–^PAX5^+^ cells in normal skin and each subset of HS skin based on presence or absence of LAs or TLSs. (**E**) Number of CD3^+^PAX5^–^ and CD3^–^PAX5^+^ cells in HS skin per unit area. (**F**) Number of CD3^+^PAX5^–^ and CD3^–^PAX5^+^ cells in each subset of HS skin based on presence or absence of LAs or TLSs. (**G**) Frequency of CD3^+^PAX5^–^ and CD3^–^PAX5^+^ cells in LA or TLS annotated regions. (**H**) Frequency of the microanatomic distribution in the dermis or subcutis of LAs and TLSs in HS skin. Data are compiled from 3 HS cases with no evidence of TLSs/LAs, 2 cases with LAs, and 4 cases with TLSs. **P < 0.01, 2-tailed Student’s *t* test.

Thirty HS cases were selected from the UCSF pathology archive for histological analysis of available hematoxylin and eosin–stained (H&E-stained) sections by a dermatopathologist. Initial evaluation of these sections revealed several structural features typically associated with disease progression, including tunnel formation, abscesses, and fibrosis (Supplemental Table 1; supplemental material available online with this article; https://doi.org/10.1172/jci.insight.169870DS1). In addition, 9 of the sections examined contained structures resembling lymphoid aggregates (LAs) or TLSs (Supplemental Figure 1A). To examine the attributes of these features in greater depth, 9 of the HS cases and 1 case of healthy skin were selected for detailed histopathologic and immunohistochemical evaluation. HS cases were subdivided into those that showed no evidence of LAs or TLSs (3 cases), cases with LAs but no discernable TLSs (2 cases), and cases with TLSs (4 cases) (Figure 1A). On H&E staining, TLSs and LAs were defined as small, discrete, well-circumscribed collections of mononuclear cells with and without a germinal center (GC) reaction, respectively. To provide supportive evidence of authentic TLSs, tissue sections were stained for complement receptor 2, also known as CD21, to highlight follicular dendritic cells (FDCs) that are normally present in GCs (Figure 1B) (15). CD21 IHC demonstrated discrete collections of FDCs in HS cases with TLSs and absence of positive staining in cases with only LAs, those with no LAs/TLSs, and normal skin.

To evaluate the distribution of the lymphocytes in HS skin, cases were stained with multiplexed CD3 and PAX5 IHC to identify T cells and B cells, respectively (Figure 1C). PAX5 does not identify terminally differentiated B lineage plasma cells that also accumulate in lesional skin (16). Digital image quantification of cells on CD3/PAX5 IHC-stained tissue sections (Supplemental Figure 1, B and C) revealed that the frequency of T cells (~80%) was greater than that of B cells (~20%) in HS skin but did not differ significantly based on the presence or absence of LAs or TLSs (Figure 1D). The accumulation of B cells in HS tissue contrasted with that of normal skin, which only showed an accumulation of T cells with less cellularity than that seen in HS skin. Across all cases, T cells were numerically more plentiful than B cells in HS skin (Figure 1, E and F). Interestingly, the number of T cells and B cells in HS skin trended toward being most sizeable in cases with no LAs/TLSs and lowest in cases with TLSs (Figure 1F). Assessment of CD3/PAX5 IHC in discrete annotated regions of LAs and TLSs showed a significant skewing toward an increased frequency of B cells in the latter (Figure 1G). Whereas LAs tended to accumulate in the dermis, inversely, TLSs tended to arise in the subcutaneous adipose tissue or areas of fibrosis in the subcutis (Figure 1H). Collectively, these results demonstrate the presence of bona fide TLSs in HS skin and show that while T cells are the predominant lymphocyte subtype, TLSs demonstrate a preponderance of B cells, thereby supporting the hypothesis that B cells may play an important role in the pathogenesis of a subset of patients with this disease.

### B cells in HS skin exhibit signatures of active maturation.

**Figure 2:**
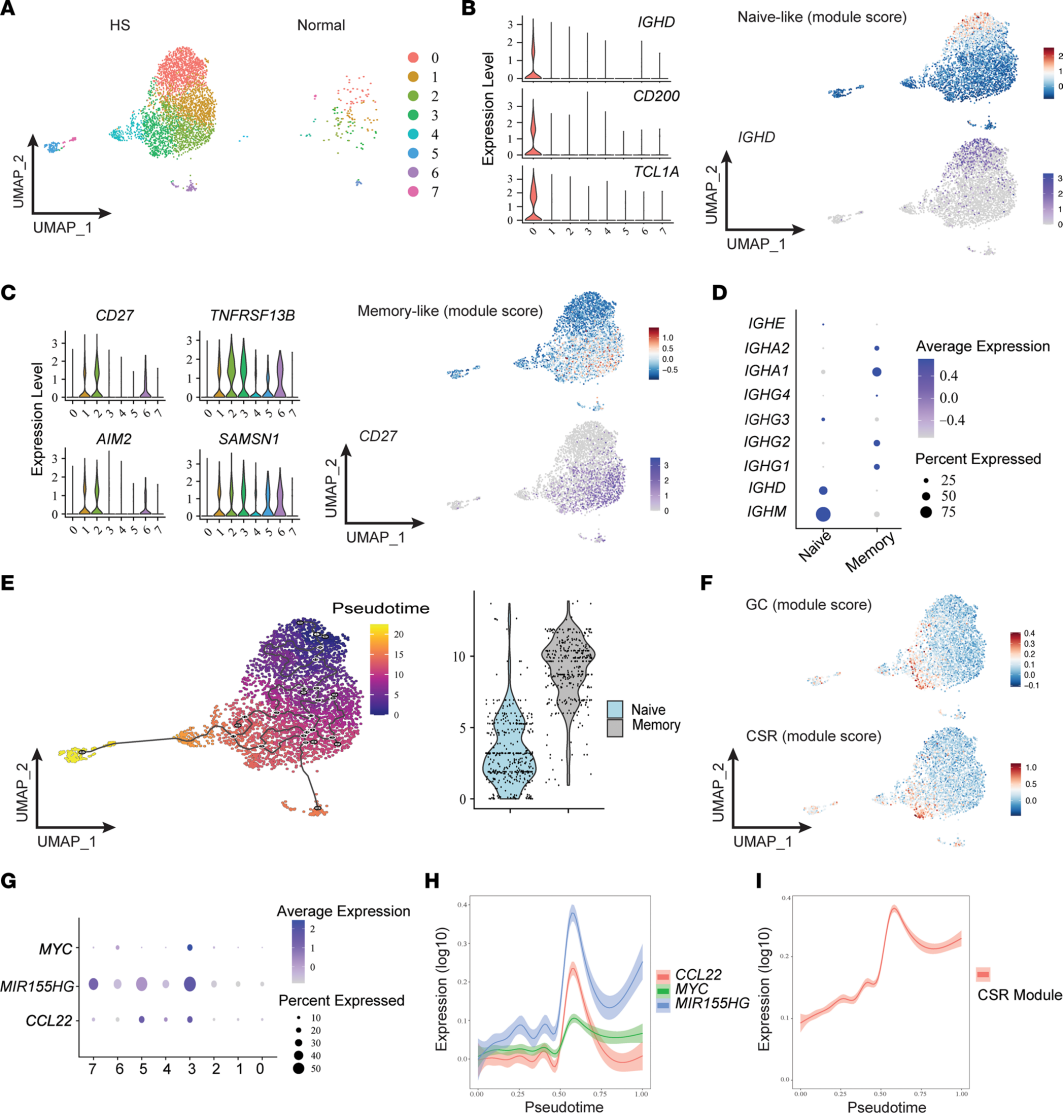
B cells in HS skin exhibit signatures of active maturation. (**A**) Uniform manifold approximation and projections (UMAPs) of B cell scRNA-Seq clusters from either HS lesions or normal skin. Data represent concatenation of samples from 5 patients with HS and 2 healthy donors. (**B**) Expression of naive B cell–associated genes and corresponding module score comprising *IGHD*, *CD200*, and *TCL1A*. UMAPs depict naive-like module score intensity and *IGHD* expression. Data represent 5 patients with HS. (**C**) Expression of memory B cell–associated genes and corresponding module score comprising *CD27*, *SAMSN1*, *TNFRSF13B*, and *AIM2*. UMAPs depict memory-like module score intensity and *CD27* expression. (**D**) Mean expression of B cell receptor isotype genes among naive and memory B cells from 5 HS lesions. (**E**) UMAP and naive versus memory comparison of B cells from 5 HS lesions analyzed with Monocle 3 for pseudotime. Black lines indicate pseudotime trajectories. (**F**) UMAP visualization of GC and CSR module scores among B cells from 5 HS lesions. (**G**) Mean expression of GC positive selection–associated genes across HS lesional B cell clusters from 5 patients. (**H** and **I**) Plots display gene expression by pseudotime.

To investigate B lymphocyte cell states in HS lesions, we performed scRNA-Seq on CD3^–^CD45^+^ cells isolated from 5 patients with HS and 5 healthy skin donors. B cells were identified by *MS4A1* expression, and their lineage was further verified based on differential presence of *CD19*, *BANK1*, *CD3E*, *GNLY*, *CD14*, *FCER1A*, and *FCGR3A* (Supplemental Figure 2A) (17). Analysis of our resulting data set divided B cells into 8 separate clusters (Figure 2A and Supplemental Figure 2A). As the number of B cells derived from healthy skin was comparatively low, we removed these cells from further analysis and focused on identifying populations present in HS lesions. In line with our purification strategy targeting CD45^+^ cells, few fully differentiated plasma cells were present in this data set (Supplemental Figure 2B) (18). In contrast, a large proportion of sequenced cells were phenotypically characteristic of naive B cells. Cluster 0 was highly enriched in cells expressing *IGHD*, *CD200*, and *TCL1A*, which identify naive cells (Figure 2B) (17). The remaining clusters were associated with activated and memory B cell populations given expression of *CD27*, *SAMSN1*, *TNFRSF13B*, and *AIM2* (Figure 2C) (17, 19). Moreover, naive cells were strongly associated with expression of *IGHM* while memory clusters exhibited preferential isotype class-switching to IgA (Figure 2D).

Analysis of patient matched blood samples revealed a broadly similar distribution of B cells (Supplemental Figure 3A). Both naive and memory populations were equivalently present in HS and normal blood samples, and these cells exhibited similar patterns of isotype switching (Supplemental Figure 3, B and C). Nonetheless, differential expression patterns of several genes implicated in B cell function were evident between HS and healthy blood cells. HS-derived blood B cells maintained lower levels of *CD83* and *CD69* while expressing elevated *CD180* and *CCR7* (Supplemental Figure 3D). The association of these genes with activation (*CD83*, *CD69*, *CD180*) (20–23) and trafficking (*CCR7*) (24) is suggestive of systemic qualitative alterations in the B cell compartment of patients with HS and consistent with B cell reorganization during inflammation (25).

Pseudotemporal ordering supported our stratification of cells based on *CD27*, *CD200*, and *TCL1A* expression. These trajectories arranged memory-like populations progressively later in the trajectories and indicated dynamic relationships between identified clusters (Figure 2E). Consistent with the presence of TLSs identified in our histological analysis, one of the B cell clusters (cluster 3) with intermediary pseudotime values was enriched for genes closely associated with GC B cells (26). In addition, this cluster exhibited preferential expression of class switch recombination (CSR) machinery genes, including *AICDA*, *APEX1*, and *XRCC5* (Figure 2F) (27). Cluster 3 B cells exhibited hallmarks of active GC positive selection. These cells expressed high levels of *MYC*, *MIR155HG*, and *CCL22*, which are important contributors to B cell affinity maturation (28–31). *CCL22* expression, in particular, overlapped closely with cluster 3 and has been previously defined as a marker of high-affinity GC B cells (Figure 2G) (29). Across pseuodotime, expression of both these GC positive selection genes (Figure 2H) and our CSR gene module score (Figure 2I) peaked at an intermediary value consistent with progressive maturation of naive cells through the GC toward terminal differentiation as plasma cells. These data demonstrate that a heterogeneous mixture of B cells spanning a spectrum of activation and differentiation cell states accumulate in HS lesions. Together, this is highly suggestive of ongoing affinity maturation within lesional skin.

### Spatial transcriptomics identifies distinct regions of TLS involvement in HS skin.

**Figure 3:**
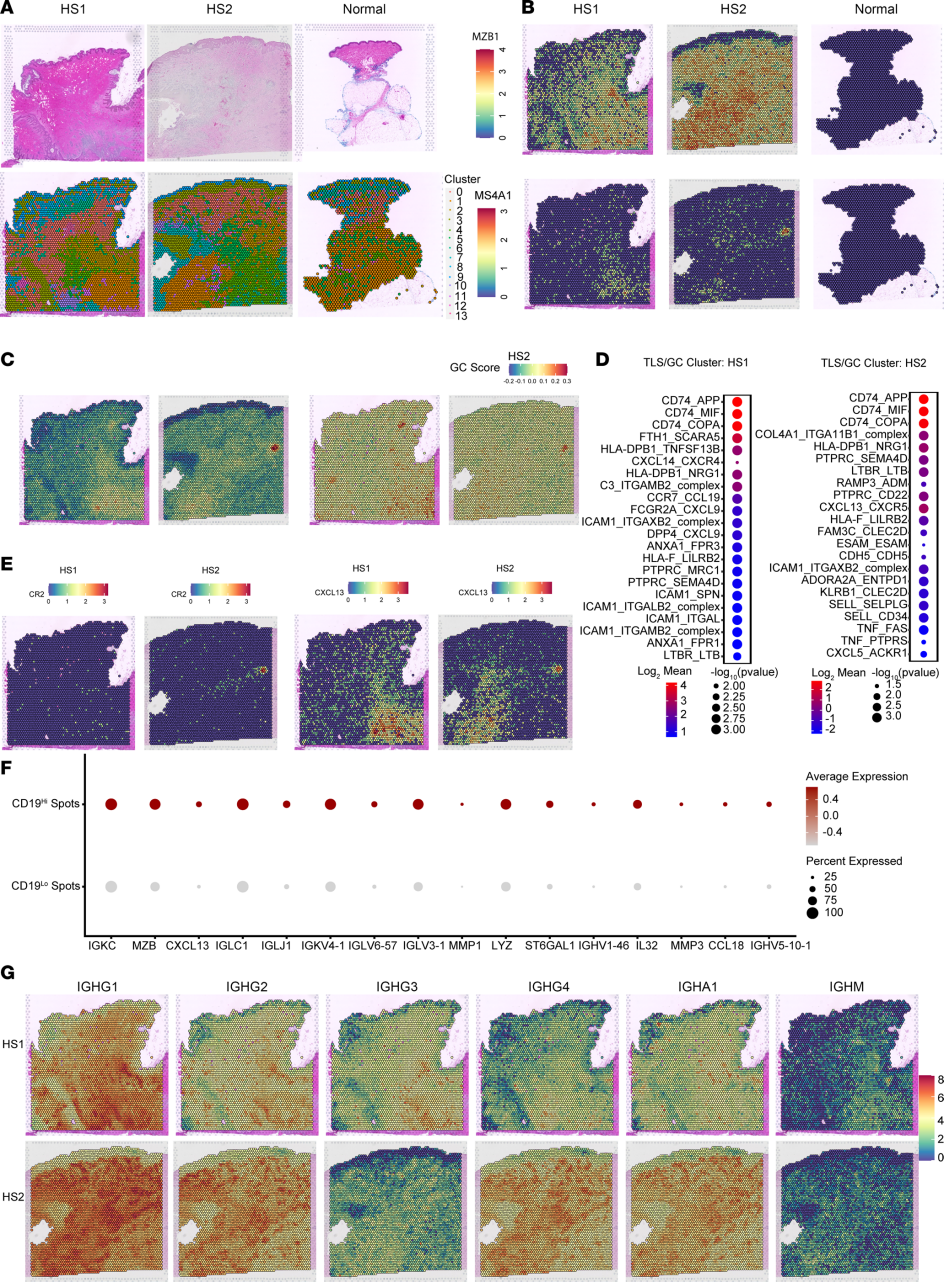
Spatial transcriptomics identifies distinct regions of TLS involvement in HS skin. (**A**) H&E staining (top) and unsupervised clustering (bottom) of spatial transcriptomics data of 2 HS samples and 1 healthy skin sample. (**B**) Spatial feature plot depicting expression of B cell lineage marker genes *MZB1* and *MS4A1* in spatial transcriptomics data of 2 HS samples and 1 healthy skin sample. (**C**) Spatial feature plot depicting module scores of TLS signature genes (left) and GC signature genes (right) in 2 HS skin samples. (**D**) Dot plot of CellPhoneDB analysis depicting the top significant ligand-receptor interactions occurring within clusters of high GC/TLS scores in HS samples (cluster 14, HS1, cluster 8, HS2) (*P* < 0.05). (**E**) Spatial feature plot depicting expression of B cell recruitment factor *CXCL13* and FDC marker *CR2* in 2 HS skin samples. (**F**) Dot plot depicting significantly increased genes comparing spots with high expression of *CD19* (*CD19* ≥ 0.5) versus low expression of *CD19* (*CD19* < 0.5) from 2 HS skin samples (adjusted *P* [padj] < 0.05, Wilcoxon test). (**G**) Spatial feature plot depicting expression of immunoglobulin genes *IGHG1*, *IGHG2*, *IGHG3*, *IGHG4*, *IGHA1*, and *IGHM* in 2 HS skin samples.

In an attempt to definitively characterize B cell organization and potential TLSs in HS, we analyzed 2 skin sections from HS lesions and 1 section of healthy skin with spatial transcriptomics (Figure 3A). Histological analysis revealed pathological changes characteristic of HS, including deep extension of epidermal tracts, widespread immune infiltration, and a histologically identified TLS in the second HS sample (HS2). While HS dermal and epidermal tissue regions clustered transcriptionally with healthy skin, regions deeper within HS lesions showed greater organizational disruption and alteration in transcriptional programming (Figure 3A). Immune cell infiltration, including B cells, was extensive in both HS1 and HS2. B cell lineage markers, *MZB1* and *MS4A1*, were expressed in both HS tissues and not detected in healthy skin (Figure 3B). Analysis with published signatures for TLS (32) and GC (26) activity showed spatially distinct areas bearing these B cell organization signatures in both HS samples, with highest activity occurring within the histologically identified TLSs (Figure 3C). Ligand-receptor interactions within the region of highest TLS/GC activity score of each HS tissue were identified with CellPhoneDB (Figure 3D) (33). Many HS1 interactions involved MHC class II genes and chemokines and were thus indicative of antigen presentation and immune cell recruitment. Antigen presentation interactions were also present within HS2; however, interactions driving B cell recruitment (CXCL13_CXCR5) (15) and GC function via CD22 engagement (34) were among the top interactions detected. While *CXCL13* expression was more widespread throughout both HS tissues, expression of *CR2*, a highly specific gene for GC organizing FDCs (35), was limited to the HS2 TLS cluster (Figure 3E). Therefore, while discrete TLS regions are readily identified in HS tissues, B cells are more broadly localized within the inflamed skin, and engagement with antigen-presenting cells may occur within and outside of TLSs.

To understand how B cells might be affecting skin tissue, we asked what transcriptional differences were increased in tissue spots containing high levels of *CD19* transcript versus low transcript signals (Figure 3F). Most differentially expressed genes were in the immunoglobulin family, with B lineage marker *MZB1* and B cell recruitment factor *CXCL13* also differentially expressed. Interestingly, several genes associated with wound repair, including *MMP1*, *MMP3*, and *IL32*, were also enriched in B cell–containing spots (36, 37). Given that immunoglobulin genes are differentially expressed within B cell–containing spots, we assessed expression of specific B cell isotypes within the tissues (Figure 3G). *IGHG1* expression was prevalent among both tissues, while *IGHG2*, *IGHG3*, and *IGHG4* expression varied among individual patients. *IGHA1*, a key mediator of mucosal tissue protection, was also highly detected in both tissues (38). In contrast, *IGHM* expression was lowest in both tissues, indicating that the majority of antibody-producing B lineage cells had class-switched. Thus, within HS lesional tissue, immunoglobulin production, potentially around areas with heightened wound repair signals, is a predominant cellular process in B cells and plasma cells.

### Fibroblasts and T cells are primed to recruit and support B cells in HS skin.

**Figure 4:**
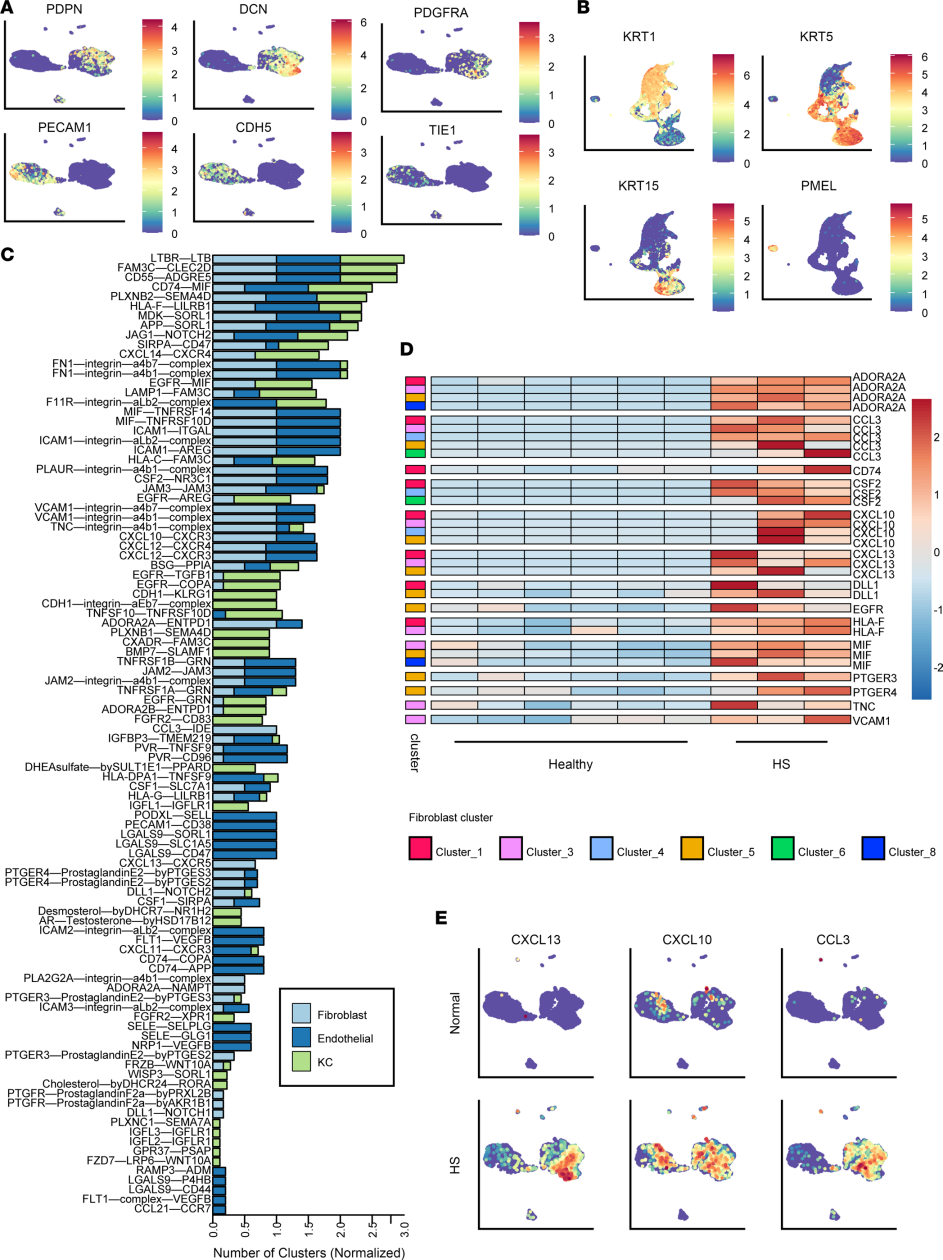
Fibroblasts are primed to support and recruit B cells in HS skin. (**A**) UMAPs of stromal cell scRNA-Seq clusters from HS lesions and normal skin showing expression of endothelial and fibroblast signature genes. Data represent samples from 3 HS donors and 6 normal skin donors. (**B**) UMAPs of scRNA-Seq data of epidermal cells from HS lesions and healthy skin showing expression of keratinocyte signature genes. Data represent samples from 2 HS donors and 2 normal skin donors. (**C**) Significant (*P* < 0.005) ligand-receptor interaction partners identified by CellPhoneDB between clusters of stromal cells (left partner) and B cells (right partner). Numbers of clusters involved are normalized to the total number of clusters involved per cell type. B cell data were utilized from 5 HS donors, keratinocyte data from 2 HS donors, and endothelial and fibroblast clusters from 3 HS donors. (**D**) Row-normalized heatmap depicting pseudobulk scRNA-Seq counts of significantly upregulated (adjusted *P* < 0.05, Wald test) genes in HS fibroblast clusters versus healthy skin fibroblast clusters. Data represent samples from 3 HS donors and 6 normal skin donors. (**E**) UMAPs of stromal cell scRNA-Seq clusters from HS lesions and healthy skin showing expression of select differentially expressed genes. Data represent samples from 3 HS donors and 6 healthy skin donors.

Next, we asked whether tissue stromal cells play a role in recruiting and supporting B cell activity in HS skin. We performed scRNA-Seq on purified CD45-negative cells from 3 HS lesional skin specimens and 6 healthy skin specimens, which clustered into subsets composed of fibroblasts (marked by *PDPN*, *DCN*, and *PDGFRA*) and endothelial cells (marked by *PECAM1*, *CDH5*, and *TIE1*) (Figure 4A). Given the potential role of keratinocytes in HS pathology, we additionally sequenced CD45-negative cells from epidermal preparations of 2 HS specimens and 2 healthy skin specimens (Figure 4B). CellPhoneDB analysis among HS skin endothelial cell clusters, fibroblast clusters, keratinocyte clusters, and B cells identified numerous interactions between stromal cell receptors/ligands and B cells (Figure 4C). Of note, *CXCL13* engagement of *CXCR5* was solely identified in fibroblast clusters. Since candidate cell interactions were widespread across numerous cell types, we then asked which stromal cell–derived interaction partners were differentially expressed between HS and healthy skin cell types. While most interaction partners expressed by endothelial cells were also expressed in healthy skin (Supplemental Figure 4A), fibroblasts in HS skin upregulated numerous molecules versus healthy controls (Figure 4D). Among these were chemokines *CXCL13*, *CXCL10*, and *CCL3* and prostaglandin receptor family members. Expression of these factors was not confined to a single fibroblast cluster and was lower or absent from healthy skin (Figure 4E). Although keratinocytes also upregulated some chemokines in HS skin, these were limited to lower abundance subsets (Supplemental Figure 4B). Therefore, fibroblasts may play a key role in recruiting and supporting B cells in HS tissues.

**Figure 5:**
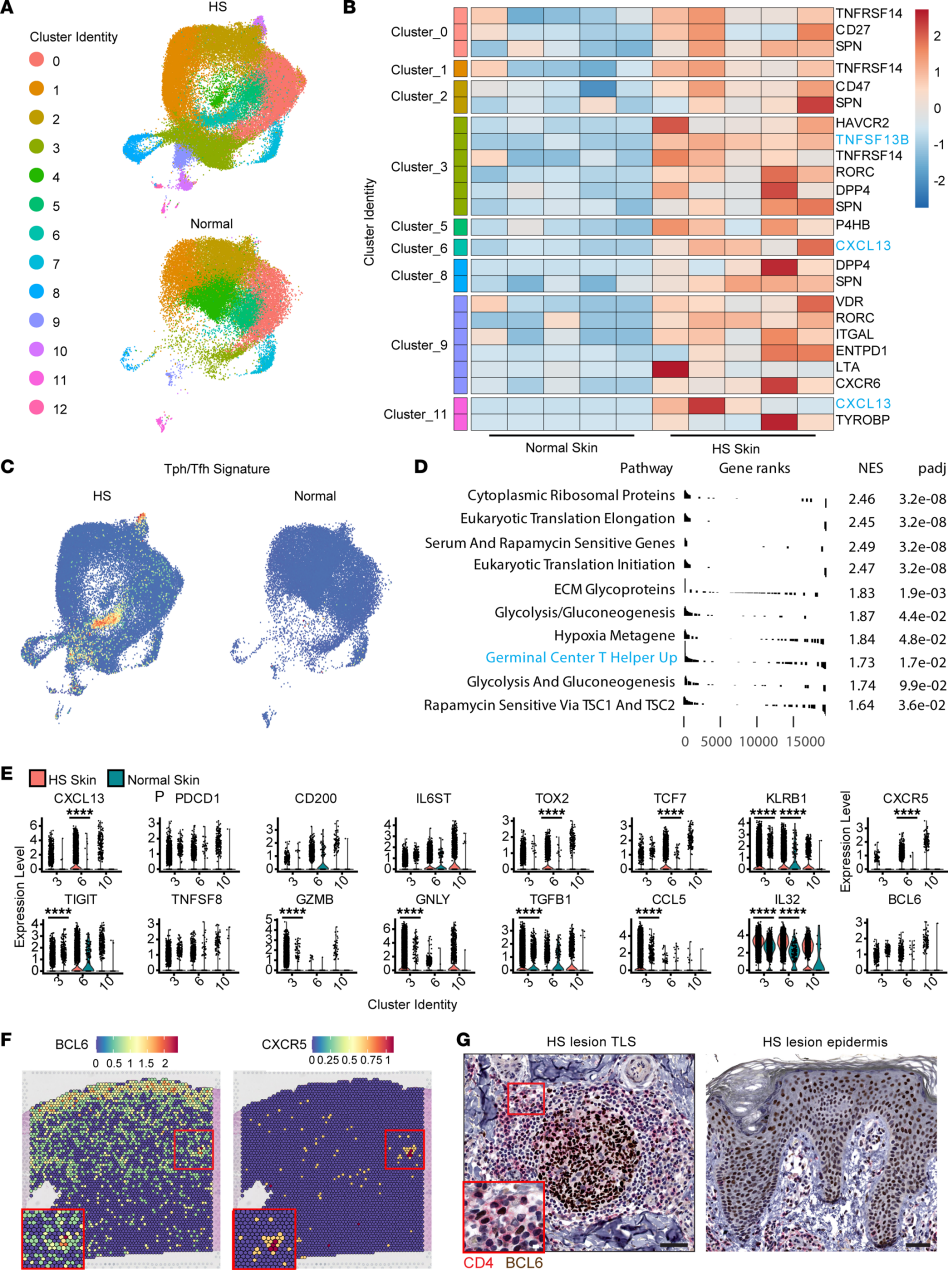
CXCL13-expressing Tph are increased in HS lesional skin. (**A**) UMAPs of CD4^+^ T cell scRNA-Seq clusters from 5 HS lesions and 5 normal skin samples. (**B**) Row-normalized heatmap depicting pseudobulk scRNA-Seq counts of significantly upregulated (padj < 0.05) genes identified as B cell interaction partners in HS CD4^+^ T cell clusters versus healthy skin CD4^+^ T cell clusters. (**C**) UMAPs of CD4^+^ T cell scRNA-Seq clusters from HS lesions and healthy skin showing expression of a Tph/Tfh gene module. (**D**) Top 10 significantly enriched (padj < 0.05) GSEA results of Human MSigDB Collections C2 gene signatures comparing cluster 6 cells from HS lesional skin with all other clusters. NES, normalized enrichment score. (**E**) Violin plots of gene expression from clusters 3, 6, and 10 from HS lesions and normal skin showing expression of select genes. Data represent samples from 5 HS donors and 5 normal skin donors. (**F**) Spatial feature plot depicting expression of *BCL6* and *CXCR5* from an HS patient lesional section with a previously identified TLS. Red box inset highlights region where a TLS gene signature is enriched. (**G**) Photomicrograph of CD4 (red chromogen)/BCL6 (brown chromogen) IHC on an HS skin section highlighting a TLS (left) and the epidermis (right). Scale bar = 50 μm; inset scale bar = 5 μm; ****P < 0.0001, Wilcoxon test.

CD4^+^ T peripheral helper cells (Tph) are critical for the formation of functional TLSs (15, 39). These cells closely resemble the T follicular helper cells (Tfh) populating lymphoid organs and promote B cell affinity maturation through provision of costimulation and supportive cytokines (39). To determine if Tph were present in HS lesions, we analyzed CD4^+^ T cell populations with scRNA-Seq (Figure 5A). CellPhoneDB analysis between CD4^+^ T cell clusters and B cells identified numerous interactions across clusters, especially *CXCL13* and the B cell survival factor *TNSF13B* (BAFF) (Figure 5B). We next generated a Tph/Tfh signature score incorporating the genes most highly expressed by these cells (40). Tph genes were highly expressed by CD4^+^ T cell cluster 6, and this population was enriched in HS samples compared with healthy controls (Figure 5C). In addition, “Germinal center T helper up” was the among the top 10 pathways significantly defined by gene set enrichment analysis (GSEA) comparing cluster 6 with all other T cells (Figure 5D) (41). Analysis of the clusters with the strongest Tph signature (clusters 3, 6, 10) revealed increased expression of canonical Tph genes, especially *CXCL13*, *TOX2* (42), *TCF7* (43), *BCL6*, *CXCR5*, and *KLRB1* (44), compared with healthy controls. Cluster 3 was also enriched in the cytotoxic genes *GZMB* and *GNLY*, which have been associated with Tph/Tfh regulation (Figure 5E) (45). The cytokine *IL32*, which has been implicated as a potential plasma cell survival factor, was enriched in both clusters 3 and 6 (Figure 5E) as well as similarly elevated in CD19^hi^ spots (Figure 3F). Consistent with the presence of Tph cells, analysis of our spatial transcriptomics data revealed enrichment of *BCL6* and *CXCR5* in the spatial region where TLS gene signatures were identified (Figure 5F). CD4 and BCL6 IHC costaining identified double-positive cells within formed TLSs (Figure 5G). Together, our analyses of the T cell and stromal compartment in HS lesions indicate that specific populations of these cells, including Tph, are expanded and actively express genes associated with TLS function and B cell maturation.

## Discussion

While B cell signatures are strongly evident in HS, the contribution of these lymphocytes to disease pathogenesis is unclear. Memory B cells and antibody-secreting plasma cells accumulate in HS lesional skin to become a prominent immunological feature (1, 4–6). Antibodies against a wide array of self-antigens are detectable in both patient sera and the skin lesions themselves, suggesting ongoing B cell activity and possible contribution to disease progression (6, 8–10). Given healthy skin maintains relatively few B cells, it is not clear how HS skin lesions support accumulation of these cells and whether specific tissue niches regulate their biological activity. Our data demonstrate the presence of TLSs, capable of supporting and shaping B cell function, within HS lesions. We find evidence of active B cell maturation and affinity maturation within lesional skin and identify a cellular niche of skin stromal constituents capable of B cell recruitment and maintenance.

TLSs are organized aggregates of immune cells that form in nonlymphoid tissues, especially under chronic inflammatory conditions. TLSs are composed of T cells, B cells, myeloid antigen-presenting cells, and supporting stromal cells coalescing to resemble secondary lymphoid organs. As such, these structures are capable of regulating B cell immune responses by facilitating T cell help and supporting formation of GC reactions (15). LAs are recognized as a histological feature of HS lesions, and the presence of fully formed TLSs has been hypothesized; however, definitive evidence of fully functional TLSs has not previously been reported (1, 4, 14). Our data demonstrate that these structures occur in advanced HS lesions. Moreover, within these TLSs, we identified evidence of active GCs and B cells expressing genes associated with affinity maturation and CSR. A substantial portion of detected cells were class-switched to IgA, which is associated with mucosal and epithelial surfaces (38). We observed that B cells accumulating in HS lesions contain population heterogeneity with evident recruitment of both naive and memory cells. Due to the limitations of our histology sample cohort, we were unable to quantify TLS frequency or identify correlations with unique patient subsets (Supplemental Table 1). Additionally, our scRNA-Seq data set comprises a relatively small patient cohort of 7 individuals with HS, with varying coverage of analyzed cell subsets (Supplemental Figure 5 and Supplemental Table 2). Therefore, it is difficult to extrapolate our data toward understanding the role of TLSs across the spectrum of disease progression, and additional work will be required to determine how these structures and associated B cell activity intersect with clinical presentation. Nonetheless, these data point to a model where B cell activity is propagated within the cutaneous tissue and accumulating lesional B cells likely mature to generate fully differentiated plasma cells in situ. This would be consistent with the relationship between TLS B cells and tissue plasma cells described in other situations of chronic inflammation. In renal cell carcinoma tumor TLSs generate IgA and IgG plasma cells that disseminate relatively large distances across the tumor tissue (46). Although we have not uncovered a direct link between HS TLS B cells and production of autoantibodies, it is plausible that cutaneous GCs give rise to the plasma cells secreting these antibodies.

Naive B cells are rare in healthy skin and therefore likely recruited to progressing HS lesions through the induction of a supportive tissue microenvironment complete with associated chemokines and maintenance factors (47). In agreement with this model, we and others have previously observed significant upregulation of the B cell survival factors *BAFF* and *APRIL* in HS lesions (4, 5). That organized TLSs are also induced by the HS inflammatory milieu provides further evidence for generation of a niche supportive of B cells during the progressive evolution of HS lesions. Our data demonstrate that several chemokines associate with B cell recruitment to TLSs, especially *CXCL13*, *CCL10*, and *CCL18*, are enriched in lesional skin (15, 32, 47, 48). Notably, fibroblasts were the only stromal cell population that expressed *CXCL13* despite the high levels of transcript detected. This indicates a key role for fibroblasts in B cell recruitment and propagation of TLS activity, consistent with previous evidence that fibroblasts mediated skin architectural changes associated with disease progression (49).

To date, limited data suggest that targeting B cells may be beneficial in the treatment of HS. B cell depletion with rituximab ameliorated HS lesions in a single patient and reduced expression of inflammatory cytokines in HS skin explant assays (12, 13). Anti–TNF-α agents are the most effective targeted biologics currently available for HS treatment (1). Notably, TNF-α is a key contributor in TLS induction, and we have previously demonstrated that ex vivo anti–TNF-α treatment of lesional skin samples reduced B cell proliferation (5, 15, 47). Other therapies relevant for B cell targeting are in development. A recent phase II clinical trial of the spleen tyrosine kinase antagonist fostamatinib reported promising clinical outcomes, highlighting the potential for treatments disrupting B cell activation and immunoglobulin production (50). Similarly, JAK inhibitors have also seen some success in early trials (51). Herein, we present findings that add to the growing evidence suggesting a role for B cells in HS pathogenesis and that immunotherapy targeting these cells may be beneficial for patients with HS. Our data imply that plasma cell depletion may be insufficient because cutaneous TLSs will be capable of replacing cells secreting pathogenic antibodies. Instead, direct manipulation of TLS formation in HS may allow for attenuation of pathogenic B cell activity while reducing systemic impacts on healthy tissues.

## Methods

### Case selection.

Thirty cases of HS were retrieved in the diagnostic and consultation files of the UCSF Dermatopathology Service and UCSF Department of Surgical Pathology. Of these, 3 cases of HS with GC reactions, 3 cases with LAs but no GC reactions, and 3 cases with no GC reactions or LAs were selected for IHC studies. Notably, 1 case initially selected as having only LAs and no GC reactions on H&E staining exhibited an FDC network on CD21 IHC and was therefore reclassified as an HS case with a TLS. One case of microscopically near-normal skin from an anogenital site was selected from UCSF Dermatopathology Service.

### Histology.

Tissue was fixed in 10% neutral-buffered formalin, routinely processed, embedded in paraffin, and stained with H&E. Formalin-fixed, paraffin-embedded sections of 4 μm thickness were stained with antibodies specific for CD21 (predilute, clone 2G9, Leica Biosystems), CD3 (predilute, clone LN10, Leica Biosystems), and PAX5 (predilute, clone DAK-Pax5, Agilent Technologies).

### Whole-slide digital image analysis.

Slides were scanned at original magnification, ×40, with an Aperio AT2 scanner (Leica Biosystems) using a 20×/0.75NA Plan Apo objective with a 2× optical magnification changer. Quantitative analysis was performed on images from sections stained with a dual antibody combination of PAX5 (red chromogen) and CD3 (brown chromogen) using QuPath (v.0.3.2) software. Small representative regions of interest of hematoxylin, 3,3′-diaminobenzidine (CD3), alkaline phosphatase (PAX5), and background were used to set stain vectors. Cells were detected using the watershed algorithm-based Detect Cells tool, using the optical density sum to segment nuclei with a cytoplasmic expansion of 1.5 μm. Cells were then classified with the Train Object Classifier tool using a neural network trained on 8–12 annotations of each cell class (CD3^+^PAX5^–^, CD3^–^PAX5^+^, and CD3^–^PAX5^–^) with the following features as input: nuclear PAX5 mean and cytoplasmic CD3 mean. LAs and TLSs were manually annotated for cell localization, and tissue area was detected with a simple threshold tool.

### Spatial transcriptomics.

Formalin-fixed, paraffin-embedded specimens from 2 lesional HS samples and 1 healthy control sample were sectioned and placed on a Visium Spatial Gene Expression Slide (10x Genomics). Probe sets were amplified and were sequenced by the Gladstone Institute. FASTQ files and the H&E-stained images were analyzed by spaceranger (v. 1.3.1) using the Visium Human Transcriptome Probe Set v1.0 (GRCh38 2020 A). The spaceranger output files were analyzed with Seurat (52). Data were normalized with SCTransform prior to principal component analysis and UMAP clustering (53). For analysis comparing multiple slides, separate slides were merged and integrated with Harmony (v. 0.1.0) (54) prior to clustering. GC and TLS scores were calculated using the AddModuleScore function using gene sets described for GCs (26) and TLSs (32) (Supplemental Table 3).

Spots with high expression of *CD19* (*CD19* ≥ 0.5) were compared with spots with low *CD19* expression (*CD19* < 0.5) using the FindMarkers command, and significantly increased genes (padj < 0.05) were plotted.

Counts assigned to clusters were exported from each HS object and analyzed with CellPhoneDB (33). The top significant interactions (*P* < 0.05) occurring within clusters of local high GC/TLS scores — cluster 14, HS1 (mean > 1), cluster 8, HS2 (mean > 0) — were identified and plotted.

### Skin processing.

We obtained 6 mm punch biopsies from 5 patients with a diagnosis of HS from an actively inflamed lesion. Healthy control skin was obtained from surgical discards and was dermatomed at 500 μm prior to processing.

Skin was finely minced with scissors and was digested overnight with 250 IU/mL collagenase IV (Worthington) and DNase 20 μg/mL (MilliporeSigma) in RPMI (Thermo Fisher Scientific) with 10% FBS at 37°C. The suspension was agitated via shaking and was filtered (100 μm, Corning) and washed before counting with a NucleoCounter NC-200 (ChemoMetec). Samples were stained for sort purification with a BD FACSAria 2 with Ghost Dye Violet 510 amine reactive dye (Tonbo), CD45 PerCP-e710 (Thermo Fisher Scientific; clone HI30), CD3 BV650 (BioLegend; clone OKT3), CD4 PE-CF594 (Thermo Fisher Scientific; clone OKT4), CD127 PE (BD Biosciences; clone HIL-7R-M21), CD25 PE-Cy7 (BD Biosciences; clone M-A251), CD45RO FITC (Thermo Fisher Scientific; clone UCHL1), CD27 APC-e780 (Thermo Fisher Scientific; clone LG.7F9), and CD8 APC (Thermo Fisher Scientific; clone OKT8). Some samples were also stained with CD235a Pacific Blue (BioLegend; clone 349108).

CD45-negative cells were sorted as singlet, Ghost Dye–negative, CD45-negative events. Some samples were also sorted as CD235a negative to exclude erythrocytes. CD3-negative events were sorted as singlet, Ghost Dye–negative, CD45-positive, CD3-negative events. Regulatory T cells were enriched from skin by sorting as singlet, Ghost Dye–negative, CD45-positive, CD3-positive, CD8-negative, CD4-positive, CD25-positive, and CD27-positive. CD4^+^ T cells not falling in the CD25-positive and CD27-positive regulatory T cell gate were also collected. Regulatory T cells and nonregulatory T cells were either spiked in a 1:1 ratio and sequenced together or sequenced on separate 10× wells.

### Keratinocyte preparation.

Discarded skin from 2 HS surgical excisions and 2 healthy skin resections was dermatomed at 1,000 μm depth and suspended overnight in a solution of 5 U/mL dispase (Stemcell Technologies catalog 07913) at 4°C. The epidermis was separated mechanically the next day and was placed in a 0.25% trypsin-EDTA solution for 15 minutes at 37°C. The epidermis was chopped with scissors and passed through a 100 μm filter, washed, and stained with anti-CD45–PerCP710 (Thermo Fisher Scientific; clone HI30), anti-CD3–BV711 (BioLegend; clone SK7), anti-CD19–FITC (Tonbo; clone SJ25C1), and Ghost Dye Violet 510 prior to sort purification with a BD FACSAria Fusion. Keratinocytes were enriched for as singlet, live, CD45-negative events.

### scRNA-Seq.

Following sort purification, CD45-negative and CD3-negative cells were quantified via nucleocounter or hemacytometer, and 10,000 to 25,000 cells were loaded per lane and sequenced with a 10x Genomics Single Cell 5′ chip. Keratinocytes (25,000 total) were loaded onto a 10x Genomics Single Cell 3′ chip. All sample loading and sequencing were performed by the UCSF Genomics Core Facility, which provided FASTQ files following sequencing.

FASTQ files were processed via Cell Ranger (v. 3.0.2) to transcriptome GRCh38 v 3.0.0 downloaded from 10x Genomics.

### Seurat analysis.

Cell Ranger–generated count files were analyzed with Seurat (4.1.0) (52). First, singlets were identified with scDblFinder (1.4.0) (55). Singlets were further selected as cells containing more than 500 nFeature_RNA and fewer than 5% mitochondrial reads. Harmony (v. 0.1.0) (54) was used for sample integration and clustering, with CD3-negative sorted cells, CD45-negative sorted cells, keratinocytes, and T cells integrated into 4 separate objects for analysis.

B cell clusters were identified from the CD3-negative Seurat object based upon *MS4A1* expression (threshold) and were subset and reclustered. B cell purity was confirmed based on the differential presence of *CD19*, *BANK1*, *CD3E*, *GNLY*, *CD14*, *FCER1A*, and *FCGR3A*. Trajectory and pseudotime analysis of B cell populations was performed with Monocle 3 (56). GC and CSR scores were calculated using the AddModuleScore function and gene sets described for GCs (26) and active CSR (27) (Supplemental Table 3).

### CellPhoneDB analysis.

CellPhoneDB (v. 4.0.0) (33) was used to identify ligand-receptor interactions between fibroblasts, endothelial cells, keratinocytes, and B cells in HS tissues or between CD4^+^ T cells and B cells in HS tissues. Normalized counts from HS clusters were exported and assessed with the CellPhoneDB statistical_analysis method. Any interaction between a B cell cluster and a keratinocyte/endothelial cell/fibroblast cluster or between a B cell cluster and a CD4^+^ T cell that was significant (*P* < 0.005) was identified. For stromal cell types, numbers of interactions between cell types were counted, normalizing for the total number of clusters identified per cell type. Differential expression analysis was performed with DESeq2 (v. 1.30.1) (57) on pseudobulk counts for individual patient clusters, comparing cells from patients with HS and healthy controls. Genes that were significantly increased in HS clusters versus controls were depicted with pheatmap (v. 1.0.12).

### GSEA.

A ranked gene list for GSEA was generated to compare expression between cluster 6 and all other HS T cell clusters. First, all HS CD4^+^ T cells were downsampled to 10,000 events. The Seurat FindMarkers command was used to determine differential expression between cluster 6 and all other clusters. To obtain rankings for all genes, the logfc.threshold and min.pct were set to 0. fgsea (v. 1.16.0) was used to determine enrichment on the MSigDB C2 Curated Gene Sets Collection. Significant (padj < 0.05) positively enriched pathways containing more than 30 genes were selected and collapsed into independent pathways with the collapsePathways command. GSEA tables for the top 10 enriched pathways were plotted.

### Statistics.

Comparisons in histological findings were assessed via 2-tailed Student’s *t* test, with *P* < 0.05 considered statistically significant. Differential expression within pseudobulked counts from scRNA-Seq data was analyzed via the DESeq Wald test, with adjusted *P* < 0.05 considered statistically significant. Statistical assessment of Seurat scRNA-Seq objects was done with the Seurat FindMarkers command using the Wilcoxon test and default settings; adjusted *P* < 0.05 was considered significant. CellPhoneDB statistical analysis was computed via the CellPhoneDB statistical analysis method, with *P* < 0.05 considered significant for spatial sequencing analysis (Figure 3). As 3 separate groups of stromal cells were assessed with CellPhoneDB in Figure 4, a more stringent *P* value (*P* < 0.005) was set. Significantly increased GSEA pathways were identified with the fgsea package, and pathways with adjusted *P* < 0.05 were considered statistically significant.

### Study approval.

The UCSF Institutional Review Board approved the proposed studies (13-11307, 19-29608, and 21-33678). All patients provided informed written consent. Keratinocytes were isolated from discarded deidentified tissue certified as Not Human Subjects Research. A list of sample origins and associated experiments is included as Supplemental Table 2.

### Data availability.

Genomic data are publicly available at GEO under the following accession numbers: GSE249793 (CD45^+^CD3^–^), GSE249622 (CD45^+^CD3^+^CD4^+^), GSE249621 (CD45^–^), GSE155850 (keratinocytes), and GSE249729 (spatial data set). All Supporting Data Values for analyses associated with the main manuscript are included in the supplement. A complete list of deidentified human tissue samples is included as Supplemental Table 2.

## Author contributions

MML, JNC, and JMM conceived and designed the study, performed experiments, analyzed data, and wrote the manuscript. MIM, SC, and JPA performed experiments and analyzed data. AEY, HBN, MP, IT, AM, HH, EK, and SLH contributed to sample acquisition and data preparation. RY analyzed data. MDR and JMM acquired research funding. All authors contributed to drafting of the manuscript.

## Supplementary Material

Supplemental data

Supplemental table 1

Supplemental table 2

Supplemental table 3

Supporting data values

## Version 1 12/19/2023

In-Press Preview

## Version 2 02/08/2024

Electronic publication
