# Supplementary material for: Tertiary lymphoid structures sustain cutaneous B cell activity in hidradenitis suppurativa
Source: JCI Insight. 2024 Feb 8;9(3):e169870. doi: 10.1172/jci.insight.169870 (PMC10967391; doi:10.1172/jci.insight.169870)
Supplement: Supplemental data [file jciinsight-9-169870-s039.pdf]

Supplement Figures:

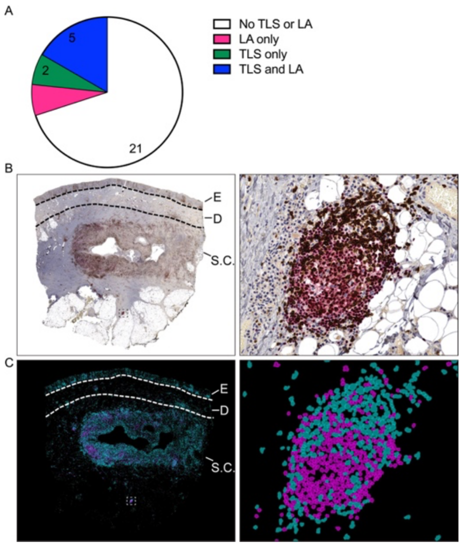

Fig S1. Digital image quantification of lymphocytes in skin. (A) Skin lesion tissue sections from 30 individual patients previously diagnosed with hidradenitis suppurativa were evaluated by a dermatopathologist for evidence of tertiary lymphoid structures (TLS) or lymphoid aggregates (LA). Numbers indicate the counts for each category. (B) Representative low power photomicrograph (left) and high power tertiary lymphoid structure (TLS; right) of the multiplex CD3 (brown chromogen)/PAX5 (red chromogen) immunohistochemical stain on HS skin. (C) Low and high power images corresponding to (B), in which the image analysis program created cell contours according to a cell segmentation algorithm and labeled cells according to signal intensity thresholds set for the red and brown chromogens. Cyan corresponds to CD3+ cells and Magenta corresponds to PAX5+ cells. E: epidermis, D: dermis, S.C.: subcutis. Dashed box indicates the location of TLS highlighted in the high power image.

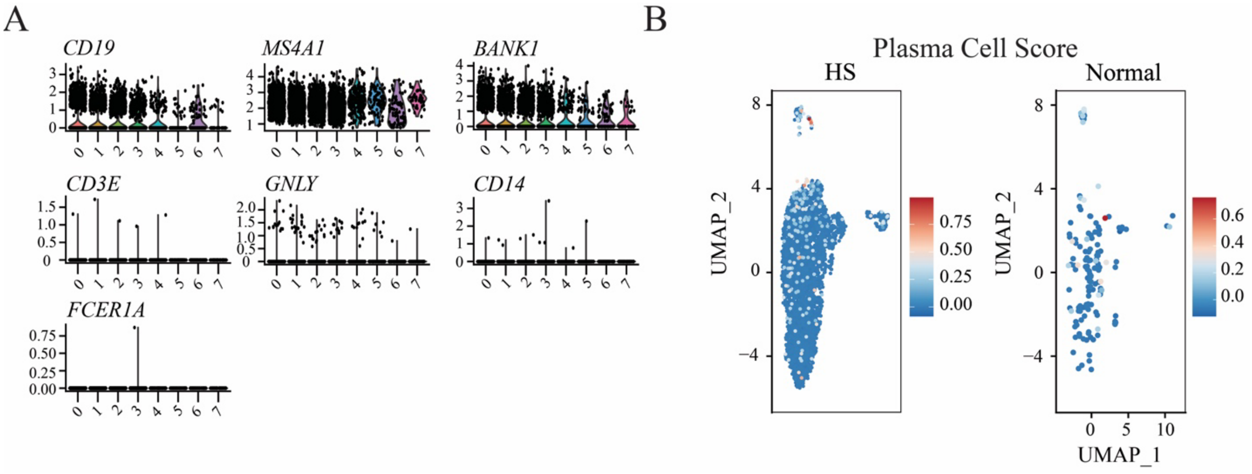

Fig S2. Single cell RNA sequencing analysis of B cells from skin. (A) Expression of lineage defining genes among *MS4A1* selected scRNA-seq cell clusters from HS lesions. (B) UMAP visualizations of plasma cell module score (*CD38*, *SDC1*, *TNFRSF17*, *MKI67*, *PRDM1*) intensity in HS lesional and normal skin B cells.

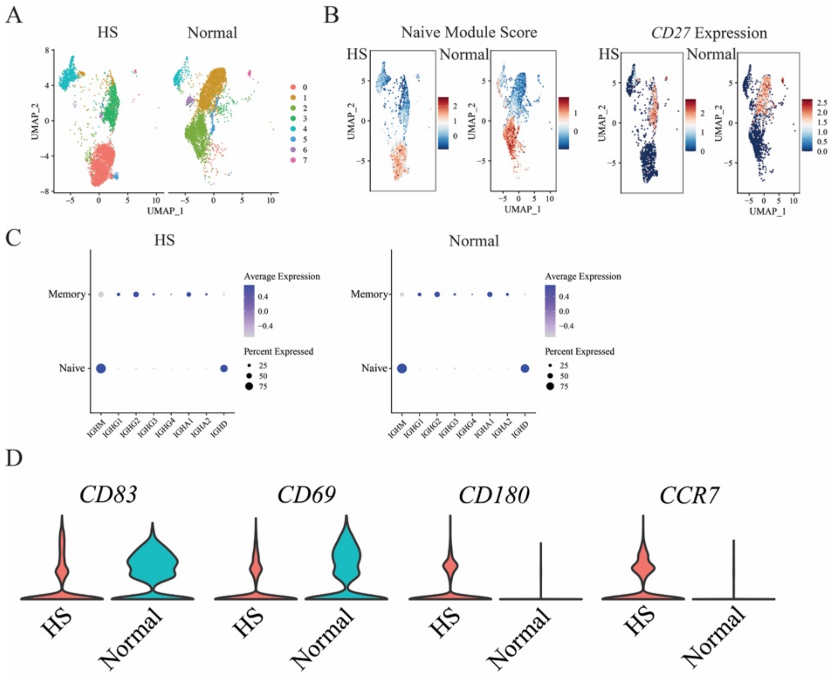

Fig S3. Comparative single cell RNA sequencing analysis of healthy and HS blood B cells. (A) UMAP projects of HS and Normal blood B cells. Data represents concatenation of samples from five HS patents and two normal donors. (B) UMAP projections depict naive-like module and *CD27* expression. (C) Mean expression of BCR isotype genes among naive and memory blood B cells from HS or healthy patients. (D) Expression of notable B cell functional genes on blood derived B cells.

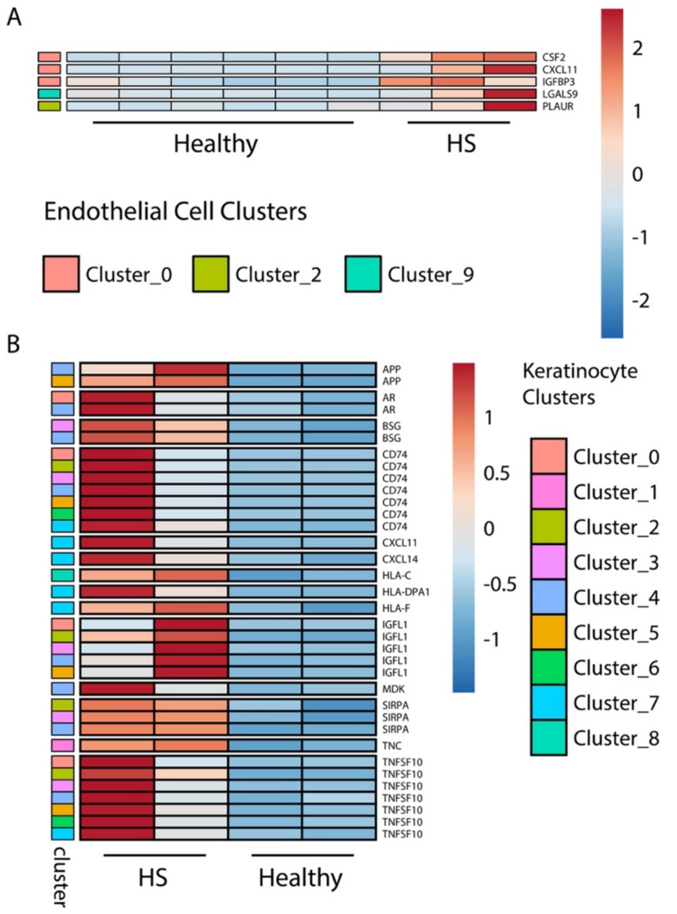

Fig S4. (A) Row-normalized heatmap depicting pseudo-bulk scRNASeq counts of significantly upregulated (adjusted $p<0.05$) genes in HS endothelial clusters versus healthy skin. (B) Row-normalized heatmap depicting pseudo-bulk scRNASeq counts of significantly upregulated (adjusted $p<0.05$) genes in HS keratinocyte clusters versus healthy skin.

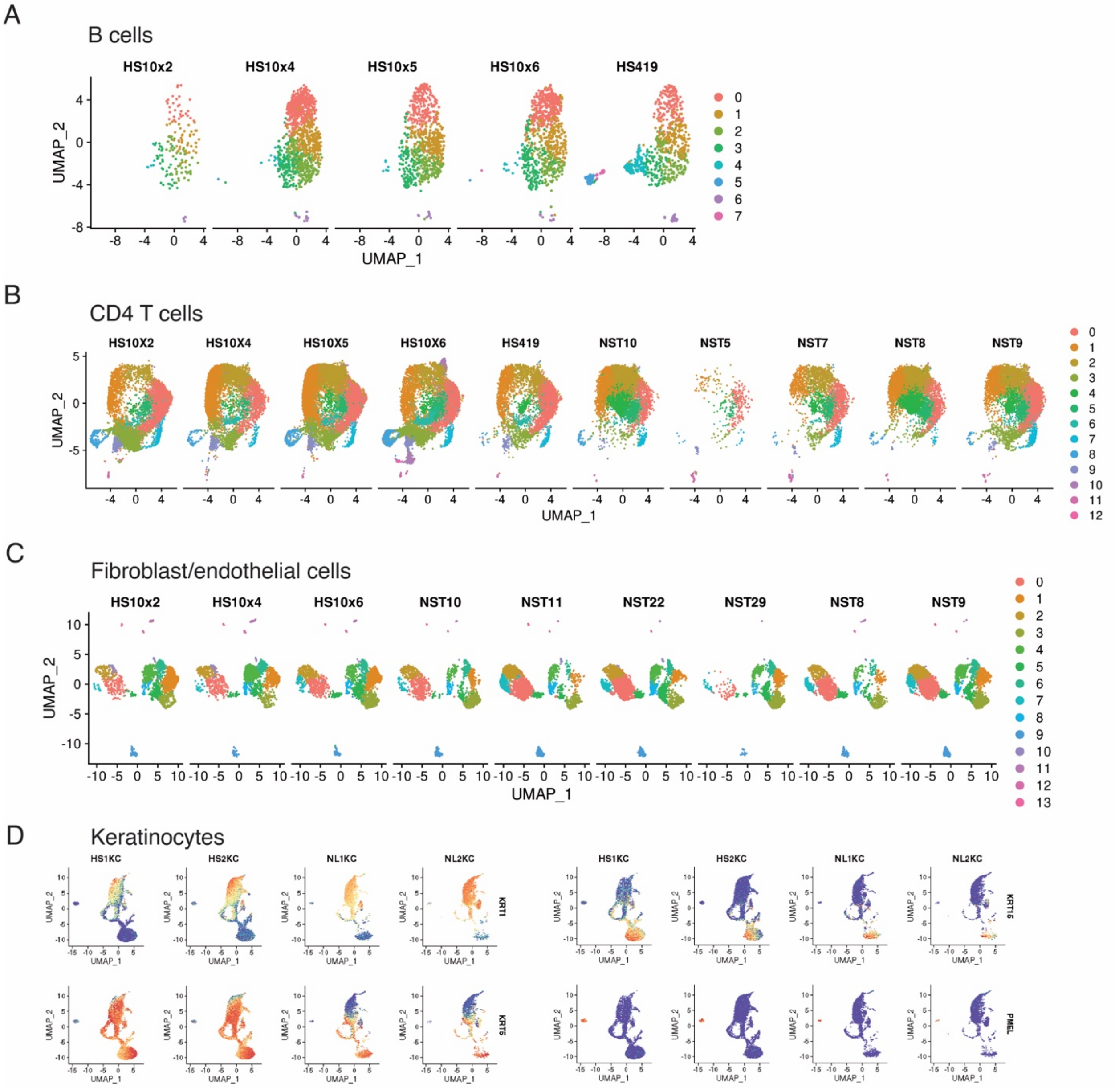

Fig S5. Patient specific UMAP projections. Each UMAP corresponds to an individual patient and is grouped by each cell type isolated: (A) B cells, (B) $CD4^{+}$ T cells, (C) Fibroblasts and endothelial cells, (D) Keratinocytes.
